# The reliability and validity of the Turkish version of the self-administered neck mobility assessment tool (s-rom-neck) in patients with chronic neck pain

**DOI:** 10.55730/1300-0144.5622

**Published:** 2023-01-31

**Authors:** Fatih ÖZDEN, Emine ASLAN TELCİ, Serbay ŞEKERÖZ, Nuray AKKAYA

**Affiliations:** 1 Division of Elderly Care, Department of Health Care Services, Köyceğiz Vocational School of Health Services, Muğla Sıtkı Koçman University, Muğla, Turkey; 2 Division of Physiotherapy and Rehabilitation, Department of Physiotherapy and Rehabilitation, Faculty of Physiotherapy and Rehabilitation, Pamukkale University, Denizli, Turkey; 3 Division of Physical Medicine and Rehabilitation, Department of Physical Medicine and Rehabilitation, Faculty of Medicine, Pamukkale University, Denizli, Turkey

**Keywords:** Neck Pain, patient-reported outcome measures, range of motion, self-evaluation

## Abstract

**Background/aim:**

The self-administered neck mobility assessment tool (S-ROM-Neck) is the subjective cervical region range of motion (S-ROM) assessment scale. The study aimed to demonstrate the reliability and validity of the Turkish version of the S-ROM-Neck in patients with chronic neck pain.

**Materials and methods:**

A cross-sectional study was conducted with a total of 60 chronic neck pain patients in the Physical Therapy and Rehabilitation Clinic of Pamukkale University Hospital between January and August 2021. The mean age of the individuals was 34.1 ± 9.9 years. Patients were assessed with S-ROM-Neck twice to prove the test-retest reliability. In addition, Visual Analogue Scale (VAS), Neck Disability Index (NDI) and bubble inclinometer measurement were used to analyze the construct validity of S-ROM-Neck.

**Results:**

The intraclass correlation coefficients of the S-ROM-Neck were higher than 0.80 (ICC > 80, CI: 0.90–98). The internal consistency of the S-ROM-Neck total score was within the acceptable limits (α = 0.754). Construct validity was high regarding the correlation analysis (r > 0.05, p < 0.01).

**Conclusion:**

Turkish S-ROM-Neck is a valid and reliable tool to assess the S-ROM of individuals with chronic neck pain.

## 1. Introduction

Cervical region range of motion (S-ROM) is a frequent evaluation method in clinical practice [1]. Several studies have demonstrated decreased S-ROM in patients with chronic neck pain [2–5]. Decreased S-ROM was mainly associated with pain (6). Therefore, S-ROM should be evaluated with an objective device and subjective patient-reported outcome measures, including pain assessment [7–9].

S-ROM is measured with various methods and tools in clinical practice (10, 11). Goniometers are sensitive to assess the S-ROM with various devices or additional tools. Wolan-Nieroda et al. demonstrated the reliability and validity of S-ROM evaluation with a head-fixed universal goniometer [12]. The universal goniometer is frequently used due to the difficulties of accessing advanced measurement methods [13, 14]. S-ROM also could be measured with an electrogoniometer, rangiometer, spirit inclinometer, liquid inclinometer, sensor-based or computerized laboratory systems [10, 15, 16]. However, there is no tool to evaluate the S-ROM subjectively (9). Clinicians can better understand the patient’s symptoms, problems, needs, and expectations with their subjective reports [9, 17].

Langenfeld et al. developed the self-administered neck mobility assessment tool (S-ROM-Neck) due to the lack of a subjective assessment tool. S-ROM-Neck is a patientreported outcome measure (PROM) to evaluate S-ROM in painful conditions [17]. To our knowledge, the Turkish version of the S-ROM is not available. The study aimed to translate and cross-culturally adapt S-ROM-Neck in Turkish. In addition, the study purposed to analyze the psychometric properties (internal consistency, test-retest reliability, construct validity) of the S-ROM-Neck in patients with chronic neck pain.

## 2. Methods

### 2.1. Participants

A cross-sectional study was conducted with a total of 60 chronic neck pain patients in the Physical Therapy and Rehabilitation Clinic of Pamukkale University Hospital between January and August 2021. Sixty chronic neck pain patients were included. Informed consent from the patients was obtained. The ethics committee of Pamukkale University approved the study protocol (No: 13.10.2020-19). The study protocol was also registered (ClinicalTrials.gov Identifier: NCT04575129).

The sample size was calculated regarding the number of items of the S-ROM-Neck. Since the S-ROM-neck includes six items, a total of 60 cases were required [18]. On the other hand, another methodological guideline suggests a minimum of 50 cases for comparative analysis studies [19].

The inclusion criteria of the study were; (1) native Turkish speakers, (2) individuals >18 years, and (3) patients with neck pain >3 months. The exclusion criteria of the study were; (1) history of neck surgery, (2) specific neck conditions or comorbidities: e.g., posttrauma, fracture, disc herniation, spasm, tumor, infection, (3) rheumatologic and neurological conditions, (4) pregnancy, and (5) cognitive problems.

### 2.2. Translation and cross-cultural adaptation process

The permission to translate the S-ROM-Neck into Turkish was received from Dr Langenfeld. International translation and cross-cultural adaptation guidelines were used [20, 21]. Firstly, two translators (native Turkish speakers and experts in German) translated the S-ROM-Neck from German into Turkish, independently. Secondly, two translations were synthesized by two academicians (experts in musculoskeletal rehabilitation). The synthesis stage included adaptation procedures regarding the Turkish linguistic features. Thirdly, a native German person (expert in Turkish) translated S-ROM-Neck back into German. The back-translated paper was compared with the original S-ROM-Neck in the fourth step. A draft version was tested in phase five. The comprehensibility of the S-ROM-Neck was pretested with a 5-point Likert-type scale in randomly selected 20 native Turkish persons. Ultimately, the final version of the Turkish S-ROM-Neck was obtained.

### 2.3. Data collection and study design

A single physiotherapist evaluated the patients in a face-to-face session. A detailed information was given to the individuals during the recruitment process.

#### Reliability

Patients were assessed with S-ROM-Neck twice to prove the test-retest reliability.

#### Validity

Visual Analogue Scale (VAS), Neck Disability Index (NDI) and bubble inclinometer measurements were used to analyze the construct validity of S-ROM-Neck.

### 2.4. Questionnaire and instruments

#### S-ROM-Neck

S-ROM-Neck includes six questions targeting the primary movements of the neck joint motion (i.e. flexion, extension, rotation, and lateral flexion). Patients were asked to report the restriction (e.g., pain, stiffness, or tension) during neck movements. S-ROM-Neck includes a visual analog scale. The left and right side of the analog scale indicates severe ROM restriction and maximum ROM, respectively. S-ROM also contains iconographic content to inform the patients about neck movements. The total score ranged from “0 to 600”. A lower score represents better ROM [9] (Table 1).

#### Visual Analog Scale (VAS)

VAS is a frequently used pain assessment tool. A minimum [0] and maximum score [100] indicate no pain and most severe pain, respectively. The validity and reliability of this scale have been demonstrated [22].

#### Neck Disability Index

NDI contains ten sections: pain intensity, personal care, weight lifting, reading, headaches, concentration, work-life, driving, sleep, and leisure activities. Each section is scored from “0” (positive status) to “5” points (negative status). A high score indicates a high disability [23].

#### Inclinometer-based S-ROM measurement

Flexion, extension, lateral flexion and rotation of the cervical joint were evaluated with the Baseline Bubble Inclinometer (Model No: 12-1056, Fabrication Enterprises, White Plains, NY). The inclinometer is valid and reliable for evaluating the S-ROM. The flexion, extension, and lateral flexion were assessed with the procedure (1). Rotation was evaluated with the procedure (2).

##### Procedure (1)

An inclinometer was placed on the top of the patient’s head as the pivot point, and the participant was asked to complete the movement to the last point and return to the initial position [24].

##### Procedure (2)

The inclinometer was placed on the forehead of the participant in the supine position, and the patient was asked to rotate. The measured value in the inclinometer was recorded. Each movement was evaluated three times separately, and the average score was noted. [24].

### 2.5. Statistical analysis

“IBM SPSS Statistics (version 25, Chicago, USA)” computer package program was used for all statistical analyses. Descriptive statistical information was given as “mean ± standard deviation (x ± SD)”, “median (IQR)” and “number, percent” (n, %). Kolmogorov–Smirnov/Shapiro–Wilk tests were used to determine the homogeneity of the data distribution. The skewness and kurtosis coefficients were considered to ensure the data normality. The intraclass correlation coefficient (ICC) was used to assess test-retest reliability. ICC > 0.80 indicates an excellent test-retest reliability [25]. Internal consistency was calculated with Cronbach’s alpha coefficient. Alpha values between 0.70 and 0.95 represent acceptable internal consistency [18]. In addition, standard error mean-standard error mean (SEM_95_) and minimum detectable change-minimal detectable change (MDC_95_) were calculated. The relationship between S-ROM-Neck and other measures was analyzed with the Spearman correlation analysis since the data was not normally distributed. A correlation coefficient >0.5 indicates excellent validity [26]. The statistical significance level was accepted as p < 0.05. The confidence interval was set to 0.05.

## 3. Results

The mean age of the individuals was 34.1 ± 9.9 years, and 51.7% (31) of the participants were women. The median (IQR) of the Neck pain duration was 12 (19) months. Characteristics and clinical data of the participants are presented in Tables 2 and 3. The S-ROM-Neck total score was 51.2 ± 4.6 (Tables 2 and 3).

### 3.1. Cross-cultural adaptation

During the cross-cultural adaptation process, no modifications were required. Besides, comprehensibility was excellent regarding the pilot study reports.

### 3.2. Reliability

The intraclass correlation coefficients of the S-ROM-Neck were higher than 0.80 (ICC > 80, CI: 0.90–98). The test-retest reliability was excellent. The internal consistency of the S-ROM-Neck total score was within the acceptable limits (α = 0.754). Besides, S-ROM-Neck’s SEM_95_ and MDC_95_ values were 6.9 and 19.3, respectively (Table 4).

### 3.3. Validity

Construct validity was high regarding the correlation analysis (r > 0.05, p < 0.01). The correlation between S-ROM-Neck with VAS, NDI, and inclinometer-based total ROM was −0.563, −0.677, and 0.904, respectively. The correlation between the S-ROM-Neck total score with inclinometer-based measurements of flexion, extension, lateral flexion and rotation ranged from 0.579 to 0.807 (Table 5). On the other hand, the relationship between inclinometer-based motion angle and S-ROM-Neck items is excellent (p < 0.01, r > 0.50) (Table 6).

## 4. Discussion

The study results proved the reliability and validity of the Turkish S-ROM-Neck. SEM_95_ and MDC_95_ values of S-ROM-Neck can provide essential reference data for patient monitoring in clinical practice. Several PROMs provide essential clinical data in patients with neck pain [27]. S-ROM-Neck is a unique questionnaire with an iconographic content. Figures enable patients to quickly understand the directions of movements (flexion, extension, right lateral flexion, left lateral flexion, right rotation, left rotation) [9, 17].

The ICC results demonstrated an excellent test-retest reliability of S-ROM-Neck (ICC > 0.80). The development study (ICC = 0.718) also reported similar results in terms of test-retest reliability. Regarding these results, the Turkish S-ROM-Neck was considered a reliable tool in different measures. Contrary to the development study, we also calculated the ICC value of each item separately [17]. The ICC values of the items were also excellent (ICC > 0.80).

Cronbach’s alpha values were within the acceptable limits both in development study and Turkish S-ROM-Neck [17]. Internal consistency result of the S-ROM-Neck demonstrated that each item examines the patient for an identical purpose. Our study also calculated item-based alpha values. These specific values proved an independent consistency of each item. Considering the items of the S-ROM (flexion, extension, right lateral flexion, left lateral flexion, right rotation, left rotation), high internal consistency was an expected hypothesis. The movement restriction direction might differ regarding the clinical status of the patient. In addition, patients may subjectively predict movement in some directions less clearly. For instance, the lowest alpha coefficient value of the S-ROM-Neck was in an inclinometer-based flexion measurement. Therefore, although standardized tools have measurement protocols, measurement properties should be strictly investigated with an alpha value.

Langenfeld et al. calculated the SEM_95_ and MDC_95_ values as 3.1 and 8.7, respectively [17]. The Turkish S-ROM-Neck’s SEM_95_ and MDC_95_ values were 6.9 and 19.3, respectively. The difference in these values may have been caused by the difference in the clinical group and the severity of the symptoms. For instance, the VAS value was approximately 35 in the development study and around 56 in our study. Therefore, The MDC_95_ value of both studies is acceptable. Clinicians can use these values as a reference value to observe the lowest detectable change when using the S-ROM-Neck measurement in the treatment follow-up of their patients. Clinicians can decide based on symptom severity for MDC_95_ reference here [28].

The higher correlation coefficient indicated the higher validity level of the S-ROM. The S-ROM-Neck was highly related to NDI, VAS, and inclinometer measurements. This result revealed a high validity of S-ROM-Neck. On the other hand, high correlations were also observed in comparing inclinometer and S-ROM-Neck item-based measurements. Considering that S-ROM measurement with bubble inclinometer, NDI and VAS are widely used, valid, and reliable gold standard neck assessment tools [22, 24, 29], our validity model revealed convergent construct validity with acceptable strength. The correlation of S-ROM-Neck with VAS was −0.31, and the correlation between NDI and S-ROM-Neck was −0.42 in the development study [17]. However, in our study, these values were found above 0.50, demonstrating higher construct validity. We attributed this situation to the symptom severity of the clinical groups rather than the efficiency of Turkish adaptation. As the severity of symptoms increases, patients can present a more responsive view to VAS and NDI values, and thus the correlation with S-ROM-Neck may be higher.

### Limitations

First, responsiveness was not analyzed since this evaluation includes a follow-up process (30). Second, our patients did not include elderly individuals. Therefore, the validity and reliability of this iconographic tool in older adults is also a matter of interest. Moreover, further research would be more potent with data obtained from other neck pathologies and different age groups. Third, S-ROM-Neck could not assess combined/complex movements than six simple neck motions (e.g., flexion, lateral flexion, rotation). Since combined movements are used in some tasks of the individual’s daily routine, more specific tools are required to holistically evaluate the S-ROM in terms of complex movements. Fourth, accurate measurement of the cervical range of motion is due to the complex and compensatory movements. Using a more reliable method, such as an S-ROM measurement device consisting of three separate inclinometers, an electronic sensor-based system, or radiography, may provide more reliable results in measuring the cervical range of motion.

#### 4.1. Conclusion

Turkish S-ROM-Neck is a valid and reliable tool to assess the S-ROM of individuals with chronic neck pain. S-ROM-Neck is a unique subjective tool with its iconographic content. Further studies should focus on responsiveness analysis of S-ROM-Neck.

## Figures and Tables

**Table 1:**
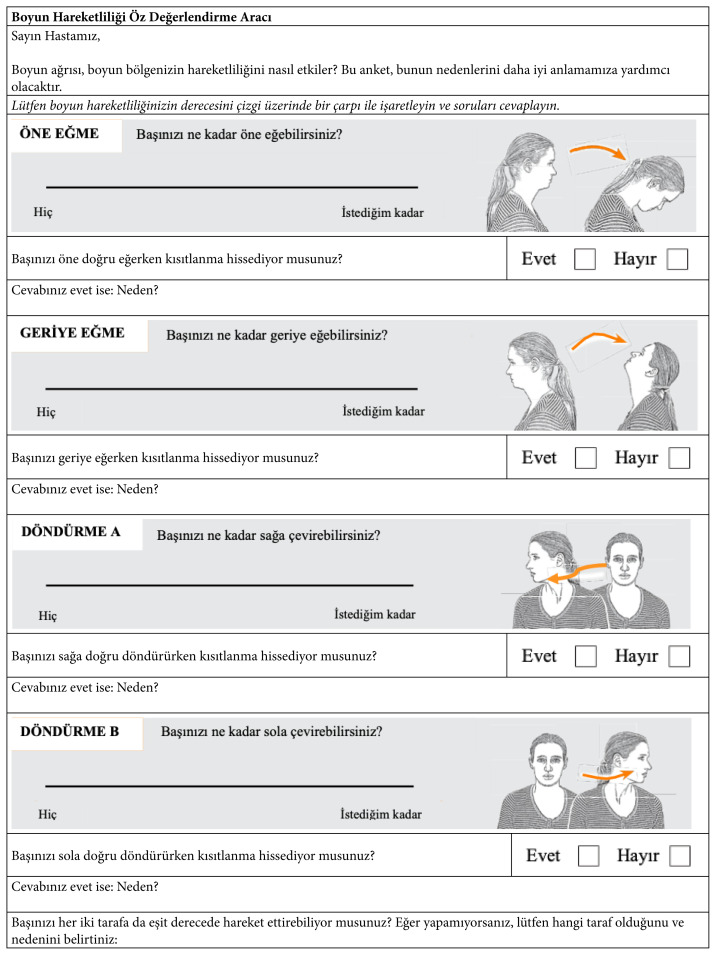

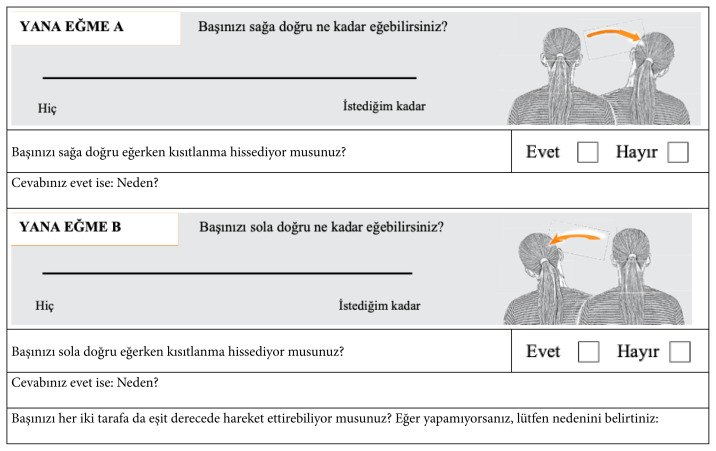
Turkish version of the S-ROM-Neck.

**Table 2:** The individual data of the patients.

n: 60	Total
Age (years, mean ± SD)	34.1 ± 9.9
Body mass index (kg/m^2^)	25.5 ± 3.4
Gender (n, %)	
Women	31 (51.7)
Men	29 (48.3)
Symptom duration (months, median, (IQR))	12 (19)
Education duration (years, mean ± SD)	11.1 ± 3.8
Marital status (n, %)	
Married	34 (56.7)
Single	23 (38.3)
Radicular pain (n, %)	
Right extremity	10 (16.7)
Left extremity	2 (3.3)
Both extremities	10 (16.7)
None	38 (63.3)
Radicular numbness (n, %)	
Right extremity	9 (15)
Left extremity	2 (3.3)
Both extremities	5 (8.3)
None	44 (73.3)

SD: standard deviation, n: number of patients, IQR: interquartile range.

**Table 3:** Mean scores of the evaluations.

n: 60		Mean ± SD	Range
VAS (mm)		56.8 ± 17.3	(23–86)
NDI (points)		12.8 ± 5.1	(5–25.5)
Inclinometer (degrees)			
	Flexion	62.3 ± 9.0	(35–75)
	Extension	61.1 ± 8.6	(38–73)
	Rotation (R)	72.5 ± 11.6	(45–87)
	Rotation (L)	74.6 ± 11.5	(39–87)
	Lateral flexion (R)	37.8 ± 4.8	(25–45)
	Lateral flexion (L)	37.8 ± 4.9	(25–48)
S-ROM-Neck (points)			
	Flexion	84.7 ± 11.3	(51–100)
	Extension	88.6 ± 10.7	(41–100)
	Rotation (R)	82.1 ± 11.9	(51–100)
	Rotation (L)	83.8 ± 11.6	(52–100)
	Lateral flexion (R)	86.5 ± 11.1	(54–100)
	Lateral flexion (L)	86.7 ± 11.9	(53–100)
	Total score	512.6 ± 46.1	(406–585)

SD: standard deviation, n: number of patients, R: right, L: left.

**Table 4:** The internal consistency and reliability of the S-ROM-Neck.

Points	Test (Mean ± SD)	Retest (Mean ± SD)	ICC (95% CI)	α	SEM_95_	MDC_95_
Flexion	84.7 ± 11.3	84.1 ± 10.7	0.943 (0.90–0.96)	0.776	2.3	6.6
Extension	88.6 ± 10.7	88.8 ± 9.6	0.958 (0.93–0.97)	0.766	2.0	5.6
Rotation (R)	82.1 ± 11.9	83.3 ± 12.7	0.963 (0.93–0.97)	0.683	2.1	5.8
Rotation (L)	83.8 ± 11.6	84.3 ± 12.3	0.942 (0.90–0.96)	0.686	2.6	7.3
Lateral flexion (R)	86.5 ± 11.1	86.9 ± 11.4	0.956 (0.92–0.97)	0.707	2.3	6.3
Lateral flexion (L)	86.7 ± 11.9	87.6 ± 10.8	0.963 (0.93–0.97)	0.671	2.1	5.8
**S-ROM-Neck (T)**	512.6 ± 46.1	515.2 ± 47.1	0.977 (0.96–0.98)	0.754	6.9	19.3

n: number of patients, ICC: Intraclass correlation coefficient, CI: Confidence interval, α: Cronbach’s alpha, SEM: Standard error of measurement; MDC: Minimal detectable change, R: right, L: left.

**Table 5:** The construct validity of the S-ROM-Neck.

n: 60	S-ROM-Neck	
	r	p
**VAS**	−0.563*	0.0001
**NDI**	−0.677*	0.0001
**Inclinometer**		
Flexion	0.723*	0.0001
Extension	0.657*	0.0001
Rotation (R)	0.807*	0.0001
Rotation (L)	0.728*	0.0001
Lateral flexion (R)	0.579*	0.0001
Lateral flexion (L)	0.620*	0.0001
Total ROM	0.904*	0.0001

* p < 0.01,

R: right, L: left, r: Spearman correlation coefficient.

**Table 6:** The construct validity of the S-ROM-Neck via inclinometer.

n: 60	r	p
**Inclinometer vs S-ROM-Neck** flexion	0.634*	0.0001
**Inclinometer vs S-ROM-Neck** extension	0.673*	0.0001
**Inclinometer vs S-ROM-Neck** rotation (right)	0.889*	0.0001
**Inclinometer vs S-ROM-Neck** rotation (left)	0.711*	0.0001
**Inclinometer vs S-ROM-Neck** lateral flexion (right)	0.826*	0.0001
**Inclinometer vs S-ROM-Neck** lateral flexion (left)	0.769*	0.0001

* p < 0.01,

r: Spearman correlation coefficient.
